# MS Annika: A New Cross-Linking Search Engine

**DOI:** 10.1021/acs.jproteome.0c01000

**Published:** 2021-04-14

**Authors:** Georg J. Pirklbauer, Christian E. Stieger, Manuel Matzinger, Stephan Winkler, Karl Mechtler, Viktoria Dorfer

**Affiliations:** † University of Applied Sciences Upper Austria, Bioinformatics Research Group, Softwarepark 11, 4232 Hagenberg, Austria; ‡ Institute of Molecular Pathology (IMP), Vienna BioCenter (VBC), Campus-Vienna-Biocenter 1, 1030 Vienna, Austria; § Chemical Biology Department Leibniz-Forschungsinstitut für Molekulare Pharmakologie (FMP), Robert-Rössle-Strasse 10, 13125 Berlin, Germany; ∥ Institute of Molecular Biotechnology (IMBA), Austrian Academy of Sciences, Vienna BioCenter (VBC), Dr. Bohr-Gasse 3, 1030 Vienna, Austria; ⊥ Gregor Mendel Institute (GMI), Austrian Academy of Sciences, Vienna BioCenter (VBC), Dr. Bohr-Gasse 3, 1030 Vienna, Austria

**Keywords:** tandem mass spectrometry, cross-linking, bioinformatics, search engine, MS/MS, XL-MS, protein-protein-interaction, PPI

## Abstract

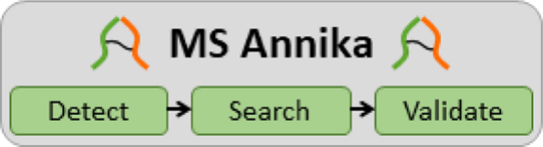

Cross-linking mass spectrometry (XL-MS) has become a powerful technique that enables insights into protein structures and protein interactions. The development of cleavable cross-linkers has further promoted XL-MS through search space reduction, thereby allowing for proteome-wide studies. These new analysis possibilities foster the development of new cross-linkers, which not every search engine can deal with out of the box. In addition, some search engines for XL-MS data also struggle with the validation of identified cross-linked peptides, that is, false discovery rate (FDR) estimation, as FDR calculation is hampered by the fact that not only one but two peptides in a single spectrum have to be correct. We here present our new search engine, MS Annika, which can identify cross-linked peptides in MS2 spectra from a wide variety of cleavable cross-linkers. We show that MS Annika provides realistic estimates of FDRs without the need of arbitrary score cutoffs, being able to provide on average 44% more identifications at a similar or better true FDR than comparable tools. In addition, MS Annika can be used on proteome-wide studies due to fast, parallelized processing and provides a way to visualize the identified cross-links in protein 3D structures.

## Introduction

Cross-linking mass spectrometry (XL-MS) allows for the identification of protein–protein interactions as well as protein structure.^1^ Until now, these two problems were tackled by individual methods that are usually expensive or time-consuming, such as NMR and X-ray crystallography.^2^ Very recently, computational approaches to estimate protein structures have shown large potential but remain to be thoroughly evaluated.^3^ With the emergence of cross-linking technology, these two areas of interest can be investigated with one technique. XL-MS builds atop MS, a field that has reliably produced high-quality scientific results for decades.^4−7^

In XL-MS, linker molecules are used to connect one or more residues of one or more proteins (usually two). There are many different types of linkers, which can be grouped into MS-cleavable and noncleavable linkers.^8^ The initially developed linkers were noncleavable linkers, realized as sturdy connections between two residues. Cleavable cross-linkers are an extension of this idea but enable cleavage of specific position in the linker molecule, allowing for increased speeds and confidence in data analysis.^1^

In both cases, the resulting mass spectra contain two peptides that must be identified to determine the parent proteins and subsequently identify the interactions thereof. The identification of single, linear peptides is a field well studied and continuously expanding. There are numerous different concepts for the search for peptides and search engines that implement these ideas.^9−13^ In this work, we focus on database search engines, but there are also other approaches for the identification of peptides such as *de novo* search and spectral library search. Standard peptide search algorithms cannot be used for the search for cross-linked peptides out of the box since they are designed to work not with multiple but with only one peptide in each spectrum.

In addition to the search engines not being able to tackle this challenge, another problem emerges: as the name suggests, a search engine traverses a database (or even all possible combinations of amino acids) for the most likely peptide sequence. This search can take a long time, depending on the number and size of proteins of interest.^10^ For data of noncleavable cross-linking experiments, this is an even greater issue as the spectrum contains two potential peptides but no clear information about the individual peptides’ masses. Therefore, all potential combinations of two peptides must be considered. By combining each potential peptide with each other potential peptide, a quadratic search space is created.^14^ For small database sizes, this problem can be solved using brute force algorithms, but as the database size increases (e.g., for proteome-wide studies), the runtime exceeds sensible time constraints even on high-performance computing clusters.

Cleavable cross-linkers aim to alleviate this so-called n-squared problem. Their ability to break apart in the mass spectrometer can cleverly be used to identify the masses of the two individual peptides. Then, the two peptides can be identified independently and reconstructed into a whole cross-link spectrum match (CSM). With the emergence of cleavable cross-linkers, new methods for measurement were developed. MS2-based approaches generally require less instrument time. MS2–MS3-based methods require more time due to the additional measurements and an MS3-capable mass spectrometer but can lead to improved results.^15−17^

Another question almost always present in proteomics MS relates to the correctness of identifications. Search engines generally identify the peptide that is most likely the correct peptide, but in some cases, the chosen sequence is wrong. The percentage of wrong identifications compared to all identifications is often referred to as the false discovery rate (FDR). To estimate this FDR, an equally sized database of incorrect potential peptides, so-called decoys, is searched simultaneously. By assuming that the number of false positives is not higher than the number of identified decoys, the result can be filtered to a desired estimated FDR.^18^ FDRs are usually calculated either by the search software or in an additional step after the search.

Due to recent advances in instrumentation and protocols, the popularity of XL-MS has steadily increased.^19^ With that, the need for new and improved algorithms is apparent.^16,20^

The question of FDR estimation is a crucial point in the development of any search tool, cross-linking or otherwise.^21,22^ As incorrect results are almost guaranteed to appear in the output, it is important to estimate their amount and filter accordingly. For cross-linking experiments, the chance of identifying at least one decoy among the two peptides is much higher than for linear peptides, and several methods to deal with this problem have been proposed.^23,24^ Multiple studies have shown that established tools often fail catastrophically to estimate true FDRs.^16,20^ Arbitrary score cutoffs and aggressive postsearch filtering steps have been suggested to come to grips with these results. However, this often obscures the method used for FDR estimation, prohibits understanding of the underlying score distribution, and removes more true positives than necessary. Therefore, the cross-linking community pushes toward more reliability and transparency in cross-linking experiments and error estimation.^21^

Identifying cross-links is helpful for numerous applications, for example, drawing of protein–protein interaction networks or mapping of cross-links to three-dimensional (3D) structures. Multiple platforms have been developed to visualize such data. One of them is xiView,^25^ an online platform that allows user upload and provides visualizations and information about the submitted data. One rather interesting functionality is the possibility of creating protein interaction networks from the data in a fully automated way. This is especially relevant for proteome-wide studies and provides a great overview of the proteins in the sample and their connections. Furthermore, xiView, provided with a protein data bank (PDB) accession number, can display the 3D model of the protein in question. The software also displays the cross-links in the 3D model and can color the links according to their length in space, which is crucial for the verification of cross-links.

In this work, we present a new search engine, MS Annika, for the identification of cross-links from tandem MS data, which is transparent in peptide identification and reliable in FDR calculation. Our algorithm is focused on MS2 spectra, alleviating the need for an additional measurement. MS Annika can deal with a wide variety of cleavable cross-linkers such as DSSO,^26^ DSBU,^27^ DSAU,^27^ DSBSO,^28^ and PIR linkers (e.g., BDP-NHP^29^), as well as cleavable zero-length cross-linkers (e.g., CDI^30^). Furthermore, MS Annika offers support for data containing ion mobilities. MS Annika is fully integrated with Thermo Proteome Discoverer (versions 2.3, 2.4, and 2.5). The fast implementation is available free of charge at https://ms.imp.ac.at/index.php?action=ms-annika and can be used to search data sets up to a proteome-wide scale.

## Methods

Our newly implemented search algorithm makes use of the typical fragmentation patterns of cleavable cross-links in the MS2 spectrum of tandem MS experiments. The alternative to this approach is to use a third MS stage (MS2–MS3 based approaches), which increases the time needed to acquire mass spectra and therefore decreases throughput. Furthermore, this approach requires a mass spectrometer with MS3 capabilities.

**Figure 1:**
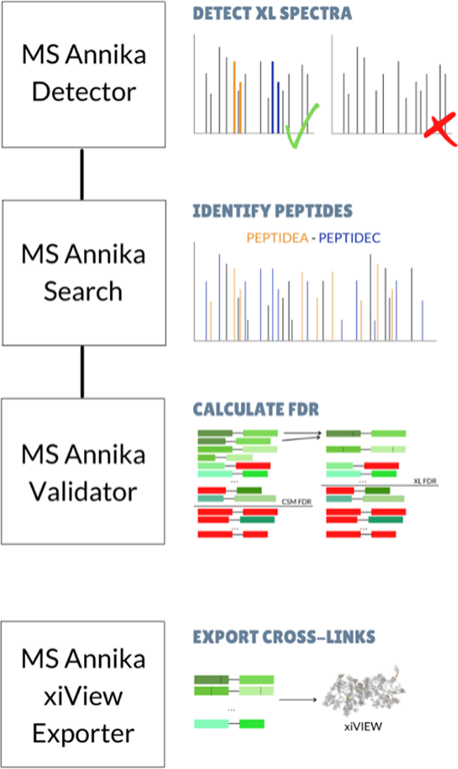
MS Annika search engine. The MS Annika Detector identifies potential ion doublets and passes the spectra to the search node if doublets are found. Spectra with identified ion doublets are searched using the MS Annika Search node. The final combination of CSM matches to cross-links as well as multilevel FDR control fall into the scope of the MS Annika Validator node. The optional xiView Exporter node can be used to export cross-links at different confidence levels, which can then be uploaded to xiView for evaluation.

It has been shown that using stepped collision energies improves the MS2 fragmentation pattern, which further decreases the requirement for additional measurements.^17^ Therefore, we focused our development effort on creating a powerful search engine that requires only MS2 data. Two crucial steps are required for the determination of cross-links from tandem MS data. First, spectra containing cross-links have to be identified. The development of cleavable cross-linkers provides a means for the identification of peptide masses from MS2 spectra. This identification is facilitated in the MS Annika Detector. The second step is to identify the cross-links from the spectra, which is implemented as the MS Annika Search Node. Finally, a validation step is often added to control the number of false positives (MS Annika Validator). An overview of the different components of MS Annika is depicted in Figure 1. A complete workflow to identify cross-linked peptides in tandem mass spectra using MS Annika in Thermo Proteome Discoverer is shown in Supporting Information Figure S1.

### MS Annika Detector: Separation of Spectra with and without Cross-Linked Peptides

Before identifying cross-links from spectra, MS Annika investigates whether a spectrum contains cross-linked peptides or not. Cleavable cross-linkers are often symmetric, with two fragmentation sites. These potential cleavage sites are positioned such that the linker breaks at an off-center position. Therefore, one peptide is attached to the heavier and one peptide is connected to the lighter side of the linker. Since multiple identical peptide pairs are contained in the sample and the linker does not always break on the same side, both peptides are ideally present with the lighter and heavier linker part. The two differently modified peptides (light and heavy) are separated by a specific mass difference in the mass spectrum, which depends on the used cross-linker. This unique information can be used to identify spectra containing cross-linked peptides^7^ In the ideal case, all four possible peaks (the two doublets) are present. These doublets are complete light-heavy peak pairs for each peptide. However, it is not always guaranteed that all four ions are present in the spectrum. In some cases, only one doublet and one ion of the other doublet are present. This method to identify spectra as cross-link spectra is named *evidence mode* since it requires ions from both peptides to be present.

To handle the remaining peptide pairs, MS Annika also offers *indication mode*. If one of the doublets is complete, while the other doublet is missing, MS Annika infers the missing peptide pair using the precursor mass. MS Annika calculates all four possible combinations (light–light, light–heavy, heavy–light, and heavy–heavy) as it is not clear if the peptides carry the light or heavy part of the linker. All pairs that fulfil the requirement of matching the precursor mass are stored for the search step. Visual representations of the different modes are shown in Supporting Information Figures S2 and S3. Spectra not containing any cross-link information can be searched with a standard database search tool. MS Amanda, for example, provides the possibility to search for dead-end links by defining cross-linker specific modifications (see Supporting Information Figure S1). Annotated spectra containing these doublet peaks are exemplarily shown in Supporting Information Figures S9 and S10.

MS Annika can also search spectra in *combined mode*, which combines indication and evidence modes to cover as many cases as possible. However, looking at more cases inadvertently increases the runtime consumption of the search process.

Additional features that are often present in the cross-linking mass spectra are the so-called diagnostic ions. These are ions that represent partial or broken cross-linkers without a peptide residue attached to it. These diagnostic ions can be deliberate (e.g., in the BDP-NHP linker) or a byproduct (cross-link residues that fragmented at the peptide connection instead of the intended fragmentation position, etc.; e.g., in DSSO^31^). Identifying such ions in a spectrum provides strong evidence that the spectrum is indeed a cross-link spectrum. The MS Annika Detector can use these diagnostic ions to identify spectra containing cross-linked peptides.

In certain cases, there is no clear isotope pattern in the MS1 spectrum, and the precursor assigned to an MS2 spectrum is wrong, representing the first isotope (1xC13). It has been shown that this problem occurs even more often with cross-linked peptides due to their higher masses.^32^ MS Annika can also handle such cases and provides two ways to search for alternative precursors: one that is based on the ions that are present in the MS1 spectrum and the one that ignores whether the ions are present in the MS1 or not and simply assumes their positions. It is possible to specify the desired mode and the number of offsets that should be considered.

### MS Annika Search: Determination of Cross-Links from Spectrum Matches

After the identification of spectra containing cross-linked peptides, the actual search step is performed. Identification of peptides in the spectrum is based on the MS Amanda search engine, which was extended to allow for searches of cross-link modifications on the individual peptides. In the search step, these modified peptide masses are used to search for each of the two individual peptides in the mass spectrum. MS Annika takes advantage of the fact that MS Amanda provides multiple peptides for each peptide mass. The top *N* peptides for each of the two peptide masses are used, where *N* is a user-defined parameter. Then, all combinations between the potential candidates for the first identified peptide mass and the candidates for the second peptide mass are created. This search results in several peptide pairs, with a combined score—the AnnikaScore—for each of the pairs. MS Annika uses the AmandaScore *S* (ref (9), eq 7) to evaluate the quality of a match of a peptide with a spectrum. The AmandaScore is a probability-based score and calculates the probability that a match happened by chance using the binomial distribution. This probability is weighted by the explained intensity based on the matched peaks and log transformed for better readability.

The AnnikaScore *A* for a spectrum *s* and two potential peptides *pep1* and *pep2* is calculated as the minimum of the AmandaScore^9^ for each of the two peptides
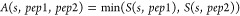


Only peptides that, together with the cross-linker mass, make up the precursor mass with consideration of some user-defined tolerance are used for the result. The two peptides are considered only if the following mass relation is fulfilled
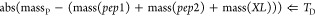
where mass_P_ is the (converted uncharged) precursor mass, mass(XL) is the mass of the cross-linker, and *T*_D_ is the detection tolerance. *T*_D_ can be defined in Da or ppm and is then converted accordingly.

We refer to two peptides identified from one spectrum as a CSM. The highest-scoring CSM, that is, the peptide pair with the highest AnnikaScore A, is selected and stored for the subsequent validation step. A step-by-step score calculation available in the pseudo-code can be found in the Supporting Information (Supporting Information S1).

### MS Annika Validator: Validation at CSM and Cross-Link Levels

A CSM represents the two identified peptides found in one spectrum. However, there are cases in which the same peptide pair appears in multiple spectra. Furthermore, one peptide can be a part of a longer peptide. In that case, CSMs of two different peptides can represent the same position in a protein. MS Annika alleviates these ambiguities in the list of CSM results by combining CSMs into cross-links, that is, a cross-link contains one or multiple CSMs that describe the same position in the protein (see also Figure 1). This method ensures that even if one of the peptides in a CSM is a substring of another peptide, these two CSMs are attributed to the same cross-link. When several CSMs merge in a cross-link, the score assigned to the cross-link is the maximum score of all included CSMs.

The final mandatory step in each experiment is the validation step. As with most experiments, using a combination of MS and database searching, a target-decoy approach to estimate the FDR is a natural choice, using a reversed database to generate decoys. A decoy hit is present when at least one of the two cross-linked peptides originates from the decoy database (see also Figure 1). MS Annika estimates the FDR at both levels at the CSM as well as at the cross-link level. For both levels, it is possible to set two thresholds for estimated FDRs. By default, these are set to 1 and 5%, respectively. To distinguish these results, MS Annika will assign a confidence to each CSM and cross-link. High-confidence results correspond to the lower threshold (e.g., 1%), and medium-confidence results correspond to the higher FDR threshold (e.g., 5%). This means filtering for results having an assigned confidence of at least “medium” provides all hits above the 5% FDR threshold. These confidences will be displayed in Proteome Discoverer and can be used to filter data to two different FDR levels without having to rerun the entire analysis, as is often the case with other tools.

In addition, MS Annika provides inter/intralink separated FDR as an additional separation technique. Intralink results are cross-linked peptides that occur on the same protein, whereas intralink results correspond to links between two different proteins. In inter/intralink separation, these two sets are validated separately, that is, a horizontal split is applied to the data set. The FDR is estimated for the two data sets individually. Then, the two data sets are reconcatenated.

### MS Annika xiView Exporter: Export-Validated Cross-Links to xiView

Visualization of identified cross-links is often essential for further validation and identification of interesting sites. xiView^25^ is a tool offering such a functionality. We therefore developed an MS Annika xiView Exporter node that can optionally be applied. This allows users to write identified cross-links to the hard drive, from where they can be uploaded to xiView.

### Evaluation

To test our novel cross-linking search engine, we applied MS Annika (version 1.2.17302 in PD 2.4) to a wide variety of publicly available data sets from different groups, some of which allow for the estimation of true FDR to compare the tool-estimated FDR, and compared the results to two other solutions, MeroX (version 2.0.1.4^14^) and XlinkX (version 1.0.0.0, for Proteome Discoverer 2.4 and 2.5^15,33,34^), the most used cross-link search engines available to our knowledge. The peptide library has also been processed with pLink (version 2.3.9^35^). .raw files were converted to .mgf files for all MeroX runs using Thermo Raw File Parser (v.1.1.11).

### FDR Estimation

To evaluate FDR estimation of MS Annika, we used two different data sets and compared the estimated and calculated FDRs.

The first data set was the peptide library by Beveridge and co-workers,^20^ which was developed as a gold standard to determine true FDRs. It contains synthetic peptides separated in multiple groups. Peptides in each group are cross-linked; groups are pooled and then measured on a Q Exactive HF-X mass spectrometer. The resulting mass spectra should contain cross-links between peptides of the same group. Cross-links between peptides of different groups are understood to be incorrect. Therefore, the true FDR can be calculated using cross-links that connect two peptides from different groups (which are therefore incorrect).

The data used in this experiment was measured using the stepped collision energy acquisition mode, which results in one MS2 spectrum containing fragments from both peptides individually, as well as the peptides themselves. Therefore, these data are well-suited to identify cross-links. The peptide library was measured twice, once cross-linked with DSSO and once with DSBU. We compared the estimated FDR to the calculated FDR of results provided by four different tools (MS Annika, MeroX, pLink, and XlinkX). To calculate the true FDR for each of the four tools, the tools were run with comparable settings (search settings are displayed in Supporting Information Table S2). pLink has been developed for noncleavable cross-linkers but can be tuned to also work with cleavable cross-linkers. For pLink, we converted the .raw file to an .mgf file using MSConvert^36^ (version 3.0.20079-3280b8471). XlinkX was run in Proteome Discoverer version 2.4 and 2.5, as in PD 2.5, and an updated FDR calculation for XlinkX was introduced. No cutoff score was used for XlinkX.

The results reported for each tool at the desired FDR cutoffs were then postprocessed using an in-house script in R to generate the figures (Supporting Information S2). For MeroX, CSMs had to be manually combined to cross-links, and for all other tools, cross-links at the desired FDR were exported. Using the identified peptide sequences, it is possible to map the group to which the peptide initially belonged back to each individual peptide. This enables the comparison of the groups for each of the two peptides. A cross-link is assumed to be correct if the two groups are the same and flagged as incorrect if they are not. One peptide (VKYVTEGMR) appears in two groups (2 and 10), and cross-links containing this peptide are assumed to be correct if the second peptide is in either one of the two groups.

The second data set used for FDR estimation was published by Ser and colleagues.^16^ In this data set, bovine serum albumin (BSA) proteins were cross-linked with DSSO and measured either mixed with a background of HEK293T cells or on their own. By searching these data against a human database containing common contaminants (CRAPome^37^), which includes BSA, it is possible to approximate true false positive rates: cross-links between residues of BSA are considered correct since they were possible in the originally cross-linked sample and cross-links that form with any other protein are considered incorrect. With this experimental setup, it is not possible to validate the BSA–BSA crosslinks, so we assume all BSA–BSA links to be correct.

We focused our analysis on the samples measured as CIDMS2-HCDMS2 acquisition mode, which can be converted for use in MS Annika using the Spectrum Grouper node in Proteome Discoverer. Due to the lack of that functionality, MeroX cannot be applied to these data out of the box, so we used the Spectrum Grouper Node (Proteome Discoverer 2.4) and exported the data as MGF files, which were then supplied to MeroX. We analyzed the data with MeroX, XlinkX for Proteome Discoverer 2.5, and MS Annika for Proteome Discoverer 2.4.

### Search Speed

As discussed briefly above, the search for cross-linked peptides can quickly become computationally expensive due to the quadratic search space expansion. This poses little to no problems in small-scale studies as the increasing power of modern compute systems can solve these problems by enumerating all possible solutions. However, for proteome-wide applications, the quadratic growth of the search space combined with a large sequence database can quickly exceed the limits of simply adding more compute power. Therefore, smart solutions are required within the software to search only a small, relevant part of the solution space.

Cleavable cross-linkers provide a crucial piece of the puzzle of determining areas of interest for the search engine. The ion doublets from the heavy and light parts of the linked peptides provide information about the peptides’ masses, thereby narrowing the search space to areas around these masses. MS Annika provides multiple different search modes [evidence mode, indication mode, and combined mode (see MS Annika Detector)], all of which are optimized for fast execution times. This allows our new search engine to process large numbers of spectra in conjunction with extensive databases in comparatively small timeframes.

To test the applicability of MS Annika to proteome-wide data, we searched a data set on *Drosophila melanogaster* embryos.^38^ We analyzed the first of three replicates consisting of just over 800,000 MS2 spectra.

In addition, we performed a runtime and memory analysis where we searched the data set by Ser and colleagues^16^ against protein databases of different sizes. We randomly sampled proteins from the Uniprot human database including isoforms combined with common contaminants, comprising approximately 42,000 proteins. The resulting FASTA files contain 1000, 5000, 10,000, 15,000, 20,000, 25,000, 30,000, 35,000, and 40,000 proteins. Searches were performed on a Virtual Machine (oVirt, Windows Server 2016, Intel Core Skylake, 2.9 GHz, 16 Cores, 80 GB RAM).

### Analysis of timsTOF Data

Trapped ion mobility mass spectrometry (tims) has recently been shown to improve the results of cross-linking experiments.^39^ However, the availability of data analysis tools that are able to tackle such data is limited. Therefore, pre- or postprocessing steps are necessary to work with these data. MS Annika works with ion mobility data out of the box, without requiring any additional preprocessing steps. In fact, the workflow in Proteome Discoverer barely changes when compared to the standard MS Annika workflow (see Supporting Information Figure S8).

We investigated the same peptide library as before, only measured on a timsTOF Pro mass spectrometer (see Data Acquisition). The resulting raw files can be imported into Proteome Discoverer and searched using a slightly adjusted workflow using the Bruker Ion Mobility Reader node, which is distributed with MS Annika and can be installed as an optional component (see Supporting Information Figure S8).

### Data Visualization

To evaluate the export functionality of the MS Annika xiView Exporter, we searched for DSSO cross-links in the data set created by Stieger and co-workers.^17^ The cross-links identified by MS Annika were exported using the MS Annika xiView Exporter and mapped to the 3D structure of *Escherichia coli* ribosomes (PDB identifier 5IT8) in xiView.

### Applicability of MS Annika to Various Cross-Linkers

To test the applicability of MS Annika to different cross-linkers, we searched several data sets using different cross-linkers other than DSSO or DSBU. We applied MS Annika to a DSAU data set (PXD018935^40^), to a BPD-NHP data set (PXD008975^41^), and to a DSBSO data set (PXD016963^42^). An overview of used data sets with corresponding linkers and PRIDE identifiers is also given in Supporting Information Table S1.

### Data Acquisition

All data used in this publication have been obtained from the PRIDE public repository (identifiers PXD008975,^41^ PXD010796,^16^ PXD011861,^17^ PXD012546,^38^ PXD014337,^20^ PXD016963,^42^ and PXD018935^40^), except data for synthetic peptide library runs on the timsTOF pro. Here, cross-linked peptides (200 ng each, DSSO or DSBU cross-linked^20^) were separated on a Dionex UltiMate 3000 HPLC RSLC nanosystem (Thermo) coupled to a timsTOF Pro (Bruker) mass spectrometer using a Captive Spray Emitter (ZDV, Bruker, ID 10 μm). Samples were loaded using a 5 μL loop onto a trap column (PharmaFluidics, μPAC C18) from where they were transferred to the analytical column (PharmaFluidics, μPAC capLC, 50 cm) heated to 50 °C. Peptides were eluted using a flow rate of 1 μL min^–1^ with the following gradient over 95 min: 0–2.5 min 1% buffer B, followed by an increasing concentration of buffer B up to 40% until min 62. This is followed by a 3 min gradient reaching 97.5% B and washing for 12 min with 97.5% B, followed by re-equilibration of the column until min 95 at 1% buffer B [buffer B: 80% acetonitrile, 19.92% H_2_O, and 0.08% trifluoroacetyl (TFA); buffer A: 99.9% H_2_O and 0.1% TFA]. Data acquisition on timsTOF Pro was performed using otofControl 6.2 based on settings as published by Steigenberger et al.^39^ with the following details: PASEF precursors were selected at *z* = 3–6 with a mobility-dependent stepped collision energy of 21.25 and 28.75 eV at an inverse reduced mobility (1/*K*_0_) of 0.73 V s/cm^2^ and 72.25 and 97.75 eV at 1.63 V s/cm^2^; collision energies were linearly interpolated between these two 1/*K*_0_ values and kept constant above or below these base points. Isolation width was set to 2 *m*/*z* at 700 *m*/*z*. Data have been deposited at the PRIDE repository with the identifier PXD022772.

## Results

We here present results obtained with our new search engine MS Annika capable of reliably identifying cross-linked peptides, suitable for a variety of cleavable cross-linkers and qualified to properly estimate the underlying FDR.

### MS Annika Provides Realistic Estimates of FDRs

**Figure 2:**
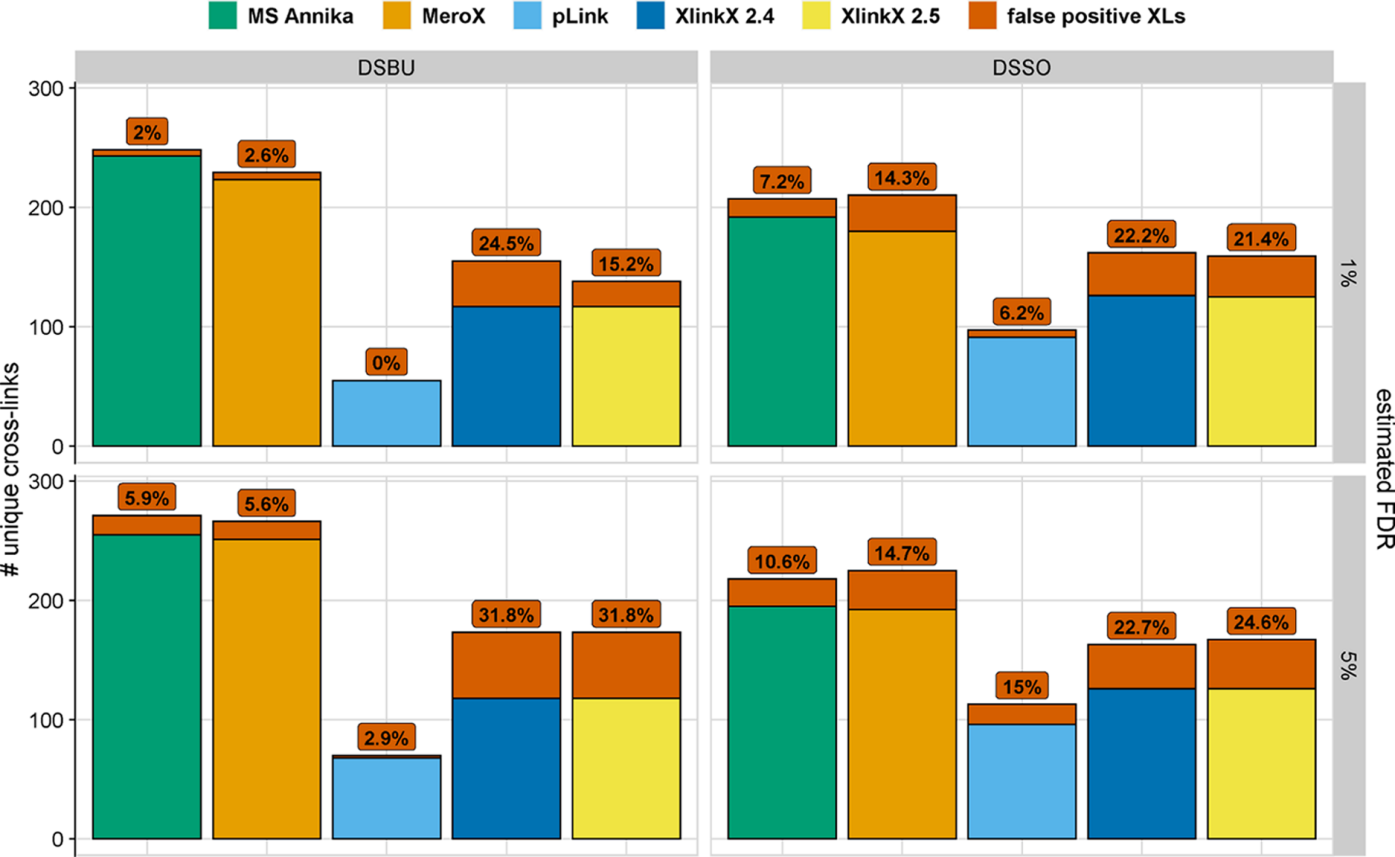
Cross-links at one and five percent estimated FDRs and calculated FDRs for four different tools, namely, MeroX, XlinkX, pLink, and MS Annika. Searches have been performed on the data set provided by Beveridge and co-workers,^20^ consisting of synthetic peptides in multiple groups, where peptides within a group are cross-linked. False-positive XLs (dark orange) are cross-linked peptides in different groups, enabling the comparison between the estimated FDR (based on a target-decoy search) and the calculated FDR based on the false-positive XLs. Calculated FDRs are generally higher than the estimated FDRs. Results shown were obtained using no score cutoff for any of the tools. For XlinkX, a minimum score difference (delta score) of 4 was set. Score cutoffs can often remove many true positives and can obfuscate the selection of cross-links. In PD 2.5, a new FDR calculation strategy was introduced for XlinkX.

As mentioned before, the peptide library can be used to compare estimated and calculated FDRs for cross-linking search engines.^20^Figure 2 shows the results for the four different tools at 1 and 5% estimated FDR and compared to the calculated FDR based on the wrongly identified cross-linkers from separate groups (given in dark orange). In all cases, MS Annika outperforms MeroX and XlinkX not only in the number of identified cross-links but also in the correctness of the estimated FDR. Only when using DSBU and 5% FDR, MeroX provides a slightly better FDR estimate (5.64 vs 5.9% of MS Annika). Still, in that special case, MS Annika provides a higher number of correctly identified cross-links (255 vs 251 of MeroX). This implies that the distribution of scores is more easily separable for MS Annika.

In PD 2.5, an updated FDR calculation for XlinkX was introduced. Still, there are massive discrepancies between the calculated and estimated FDRs. pLink provides a better FDR estimation at 1% FDR (0% for DSBU and 6.2% for DSSO) but provides a significant lower number of identified correct cross-links than all other tools.

MS Annika performs similar when looking at CSM level FDRs. At 1% estimated CSM level FDR, the calculated FDR for the DSBU data set is 1.45, and for DSSO, it is 2.5 (see Supporting Information Table S3). For the DSSO data set, the overlap of identified cross-links within the same group among all four search engines is relatively high (70 cross-links, Supporting Information Figure S4), whereas for DSBU, MS Annika and MeroX agree on a higher number of identifications (207 cross-links, Supporting Information Figure S5).

**Figure 3:**
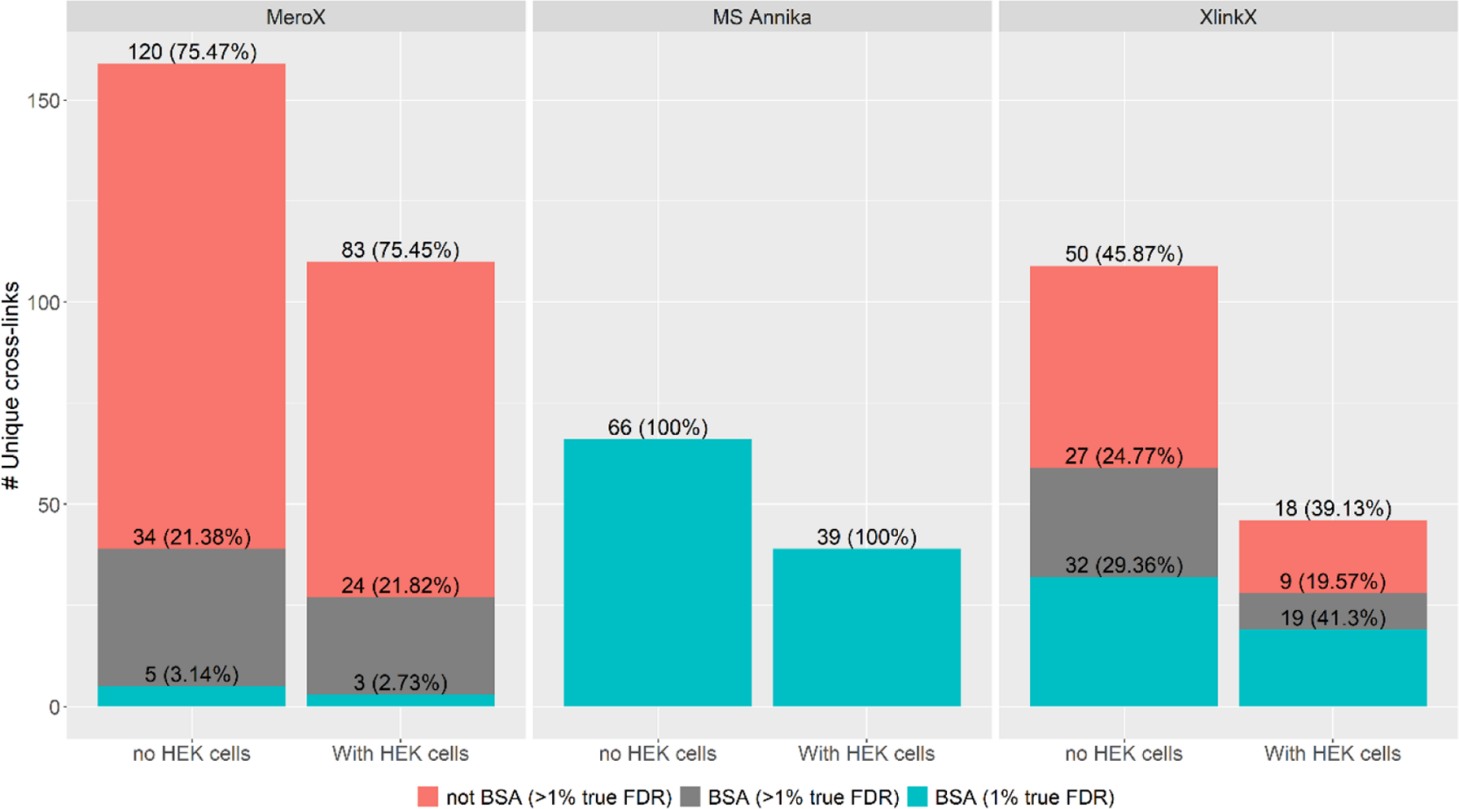
Unique cross-link counts for measurements from the CIDMS2-HCDMS2 acquisition mode of the data set created by Ser et al.^16^ All three tools were set to 1% estimated FDR without additional score cutoffs. While MeroX and XlinkX both provide more results, large fractions of identified cross-links are non-BSA cross-links (red). When applying a 1% postsearch score cutoff (removing the lowest-scoring item until 1% or less are incorrect items), only the blue items are retained. MS Annika properly separates true and false positives and provides only correct identifications.

Figure 3 shows results for the data set published by Ser and colleagues,^16^ where cross-linked peptides originating from BSA proteins can be considered as correct, and cross-links with non-BSA proteins are considered to be incorrect. Strikingly, MS Annika is the only tool that provides a true FDR of 1% at an estimated FDR of 1%. For MeroX, 75% of identified cross-links are not connecting BSA residues, while 46 and 39% of all identifications are non-BSA cross-links in the XlinkX results. We confirmed that the score cutoff of 100 for XlinkX proposed by Ser and co-workers corresponds to 1% true FDR (blue bars). Items in gray are BSA cross-links that would be removed by applying the 1% true FDR cutoff. In all of the above cases, a single incorrect cross-link is enough to exceed the 1% FDR cutoff, highlighting the importance of strong separation between true and false positives.

### MS Annika Can Tackle Proteome-Wide Studies

To ensure MS Annika’s applicability to large data sets, we analyzed a data set comprising more than 800,000 MS2 spectra of *D. melanogaster* embryos.^38^ The parallelized architecture of MS Annika takes advantage of multiple processing cores on a processor and finished the workload in 50 h (16 Cores, 2.9 GHz, 80 GB RAM). Our software is well-suited to cluster-compute environments but works just as well on desktop PCs. MS Annika was able to identify 3983 CSMs, which result in 1902 unique cross-linked residues, each at a respective FDR threshold of 5%.

Benchmarking MS Annika against a set of randomly sampled protein databases of different sizes revealed a logarithmic runtime and memory behavior as depicted in Supporting Information Figure S6. This fits our time complexity analysis when investigating the code, which in the worst case corresponds to *O*(*n* × *m*), where *n* is the number of considered doublet pairs and *m* is the number of spectra.

### MS Annika Can Identify Cross-Linked Peptides from Trapped Ion Mobility

Cross-linking experiments can benefit from the usage of tims.^39^ We investigated the peptide library described above on a timsTOF Pro mass spectrometer (see Methods). MS Annika was able to quickly identify 165 unique cross-links at 1% estimated FDR for DSSO cross-linked data, with a true FDR (calculated as described above) of 3% (160 true and five false positives). At a more relaxed 5% estimated FDR, MS Annika identified 16 false and 167 true positives for a true FDR of 8.8%. For DSBU, 185 unique cross-links could be identified at 1% estimated FDR, with a true false positive rate of 3.8% (178 true and 7 false positives). At 5%, 22 false and 183 true positives were identified, with a true false positive rate of 10.7%. Due to the early stages of cross-linking paired with ion mobility mass spectrometry, these results will undoubtedly improve as technology and methods are developed further.

### MS Annika Provides an Interface to Easily Export and View Cross-Links as Protein Interaction Networks as Well as 3D Structures

**Figure 4:**
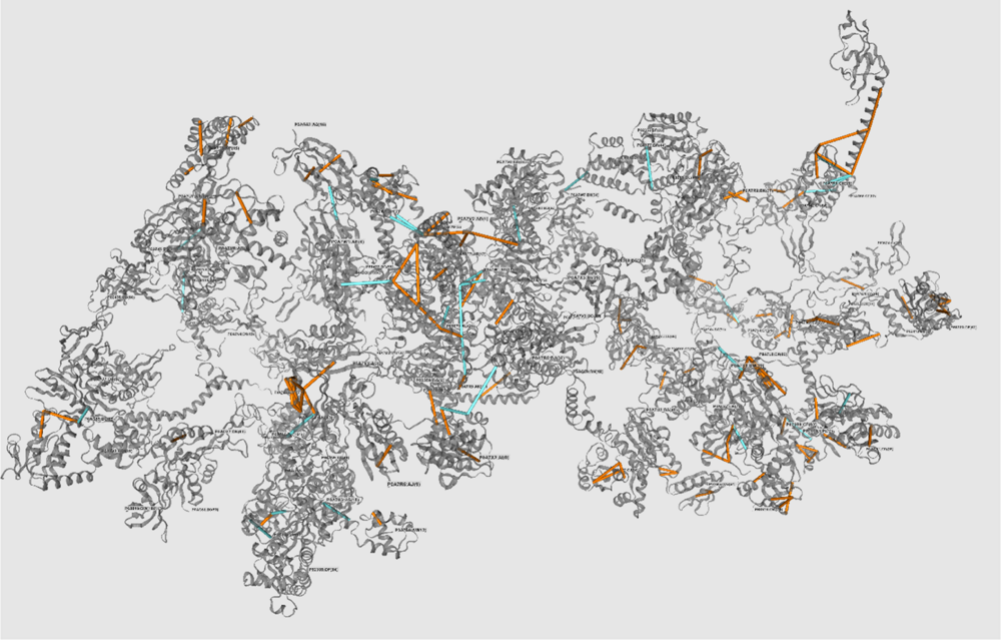
Visualization in xiView of DSSO cross-links in the data set created by Stieger et al.^17^ and identified by MS Annika mapped to the 3D structure of *E. coli* ribosomes (PDB identifier 5IT8), displaying only cross-links shorter than 26 Å. Orange cross-links connect two residues of the same protein (intralinks), while blue cross-links span residues between different proteins (interlinks).

Visualizing identified cross-links is a great way to investigate and validate results, for example, by creating protein interaction networks from the data in a fully automated way (for an example see Supporting Information Figure S7). Figure 4 shows the 3D structure of protein 5IT8 in xiView^25^ with DSSO cross-links identified using MS Annika and exported using MS Annika xiView Exporter. Links are colored according to their length in space, and only cross-linkers shorter than 26 Å are displayed. xiView was able to map 180 cross-links to the structure, with 134 of them satisfying the distance constraint. For additional 253 cross-links, at least one peptide was identified on the structure. In total, more than 70% of identified cross-links can be connected to the 3D structure.

### MS Annika Can Deal with a Wide Array of Different Cross-Linkers and Data Sources

The method of setting up linker molecules in Proteome Discoverer and extended by settings in MS Annika allows for the definition of a wide array of different MS-cleavable cross-linkers. Table 1 gives an overview of all cleavable cross-linkers MS Annika can deal with. We have successfully used MS Annika with DSSO, DSBU, DSAU, DSBSO, and BDP-NHP linkers, and identified CSMs and cross-links at 1 and 5% FDR are given in Supporting Information Table S1. MS Annika should, however, be able to deal with any linker with the same functional principle, for example, sulfoxy-based linkers, urea linkers and PIR linkers. With the expansion of the modification editor in Proteome Discoverer 2.5, it is also possible to define zero-length cleavable cross-linkers (e.g., CDI).

**Table 1:**
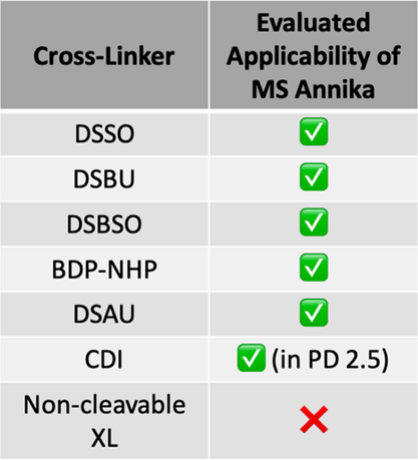
Evaluated Applicability of MS Annika to Data Sets Measured Using Various Cross-Links^a^

a Results for these evaluations can be found in Supporting Information Table S1. The application to CDI linkers is only available in MS Annika for Proteome Discoverer 2.5. In addition to the mentioned linkers, MS Annika can deal with any sulfoxy-based, urea or PIR linker.

## Discussion and Conclusions

We have developed a new cross-linking search engine, called MS Annika, that can deal with a wide variety of cleavable cross-linkers. We have tested our search engine to work with DSSO, DSBU, DSAU, BPD-NHP, or DSBSO linkers, but it can in theory deal with any (novel) cleavable linker. The focus of the search engine lies on the reliable and fast identification of CSMs and cross-links of cleavable cross-linkers from MS2 data only. MS Annika uses the MS Amanda search engine to identify each of the two cross-linked peptides in a mass spectrum. The final Annika score is the minimum Amanda score of the two peptides. Taking the minimum score of the two identified peptides rather than, for example, a geometric mean, penalizes CSMs that have one very high-scoring identification and one with a rather low score, which would otherwise add potentially wrong identifications to the result.

In this work, we have shown that MS Annika can outperform other search engines on the tested data sets, both in the number of identifications as well as in the correctness of results. For data sets where a real FDR can be calculated and compared to the estimated FDR, MS Annika is able to provide a better FDR estimation than other tools, such as XlinkX or MeroX. Interestingly, these estimates are closer to the truth for DSBU data, whereas all tools perform significantly worse when estimating FDR for DSSO data. Estimating FDRs on small data sets, such as the peptide library or the BSA data set used in this manuscript, is of course very fragile as a single false identification can easily hamper the estimation. For cross-linking experiments, this is even worse as always two peptides must be considered. Still, this is also true for the DSBU data where this phenomenon did not emerge. This discrepancy has to be further investigated but just might be dependent on the fine tuning of the used settings or the data set quality.

In this manuscript, we also show that MS Annika can tackle proteome-wide studies and export results to validate cross-links in protein 3D structures using xiView. In addition, the integrated workflow allows not only for Thermo raw data but also trapped ion mobility data from Bruker instruments using the Ion Mobility Reader node provided by the Institute of Molecular Pathology, Vienna (included with the MS Annika installer).

## Availability and Limitations

MS Annika is implemented in C# as nodes for Thermo Proteome Discoverer (2.3, 2.4, and 2.5) and available free of charge at https://ms.imp.ac.at/index.php?action=ms-annika. It is currently limited to only work with cleavable cross-linkers. A standalone version is in development that is being implemented in .NET Core and .NET Standard to ensure operating system independent usability and includes a developer documentation. Pseudo-code documenting the functionality of the main features of MS Annika are available in Supporting Information S1. The license information and a detailed user manual including parameter descriptions and a step-by-step instruction with sample files, how to run MS Annika, and how results look like are also available on the homepage. System requirements are similar to requirements of Proteome Discoverer, and we were able to run it without problems on a desktop machine (Win10, Intel Core i5, 4 Cores, 3.20 Ghz, 16 GB RAM). Data sets comprising more than a million spectra will still run on such a system; however, it will definitely take a significant amount of time.

All data sets used are freely available. Data for the timsTOF measurements of the synthetic peptide library have been deposited on the PRIDE repository (https://www.ebi.ac.uk/pride/archive/, PXD022772).

## Supporting Information Available

MS Annika Workflow in Proteome Discoverer; evidence mode of MS Annika; indication mode of MS Annika; numbers of identified cross-links and CSMs for different data sets presented in this work; settings for different tools mentioned in the main text; CSM level FDRs for MS Annika; overlap of cross-linkers for DSSO-linked peptides; overlap of cross-linkers for DSBU-linked peptide; runtime and memory analysis; protein interaction network created within xiView; modified workflow for timsTOF data using the Bruker Ion Mobility Reader; example CSM of the DSSO data set by Beveridge et al.^20^ as depicted in Thermo Proteome Discoverer 2.5; and example CSM of the DSSO data set by Beveridge et al.^20^ as depicted in Thermo Proteome Discoverer 2.5 (PDF)
Pseudo-code of the main functionality of MS Annika (MSAnnika_pseudo_code.pdf) (PDF)
In-house R script used to create Figure S2 (MSAnnika_plots_bar_and_venn.R) (TXT)
