# Supplementary material for: MS Annika: A New Cross-Linking Search Engine
Source: J Proteome Res. 2021 Apr 14;20(5):2560–9. doi: 10.1021/acs.jproteome.0c01000 (PMC8155564; doi:10.1021/acs.jproteome.0c01000)
Supplement: Supplementary file 1 — pr0c01000_si_001.pdf [file pr0c01000_si_001.pdf]

MS Annika: A new Cross-Linking Search Engine
Supplemental Information

Georg J. Pirklbauer [a], Christian E. Stieger [b, c], Manuel Matzinger [b], Stephan Winkler [a], Karl Mechtler [b, d, e] and Viktoria Dorfer [a*]

[a] University of Applied Sciences Upper Austria, Bioinformatics Research Group, Softwarepark 11, 4232 Hagenberg, Austria

[b] Institute of Molecular Pathology (IMP), Vienna BioCenter (VBC), Campus-Vienna-Biocenter 1, 1030 Vienna, Austria

[c] Chemical Biology Department Leibniz-Forschungsinstitut für Molekulare Pharmakologie (FMP), Robert-Rössle-Strasse 10, 13125 Berlin, Germany

[d] Institute of Molecular Biotechnology (IMBA), Austrian Academy of Sciences, Vienna BioCenter (VBC), Dr. Bohr-Gasse 3, 1030 Vienna, Austria

[e] Gregor Mendel Institute (GMI), Austrian Academy of Sciences, Vienna BioCenter (VBC), Dr. Bohr-Gasse 3, 1030 Vienna, Austria

* Corresponding Author: Viktoria Dorfer, viktoria.dorfer@fh-hagenberg.at, 0043 50804 22740

Table of Contents

Supplementary Figure 1: The MS Annika Workflow in Proteome Discoverer

Supplementary Figure 2: Evidence Mode of MS Annika

Supplementary Figure 3: Indication Mode of MS Annika

Supplementary Table 1: Numbers of identified cross-links and CSMs for different data sets presented in this work

Supplementary Table 2: Settings for different tools mentioned in the main text

Supplementary Table 3: CSM level FDRs for MS Annika

Supplementary Figure 4: Overlap of cross-linkers for DSSO linked peptides

Supplementary Figure 5: Overlap of cross-linkers for DSBU linked peptide

Supplementary Figure 6: Runtime and memory analysis

Supplementary Figure 7: Protein interaction network created within xiView

Supplementary Figure 8: Modified Workflow for timsTOF data, using the Bruker Ion Mobility Reader

Supplementary Figure 9: Example CSM of the DSSO data set by Beveridge et al. [20], as depicted in Thermo Proteome Discoverer 2.5.

Supplementary Figure 10: Example CSM of the DSSO data set by Beveridge et al. [20], as depicted in Thermo Proteome Discoverer 2.5.

Supplementary Material 1: Pseudo-code of the main functionality of MS Annika (MSAnnika_pseudo_code.pdf)

Supplementary Material 2: In-house R script used to create Figure 2 (MSAnnika_plots_bar_and_venn.R)

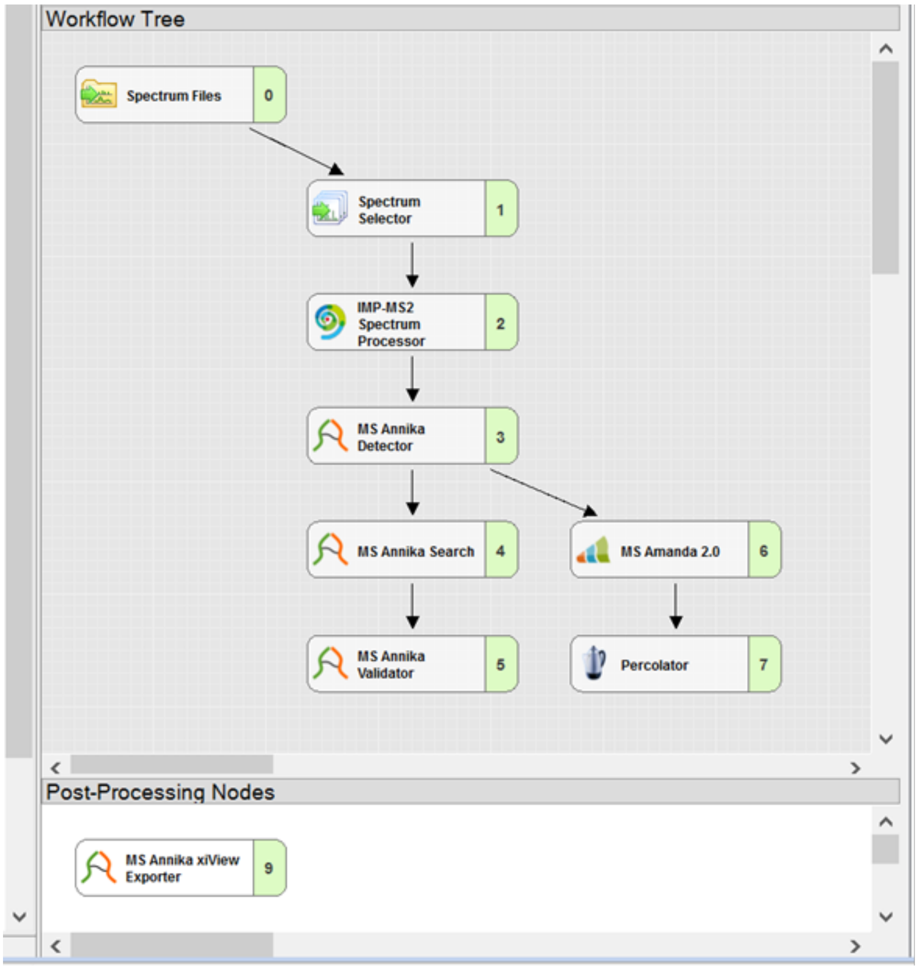

Supplementary Figure 1: The MS Annika Workflow in Proteome Discoverer. Spectra are read from a variety of different formats using the Spectrum Files node. The Spectrum Selector can be used to filter spectra of interest. The optional IMP-MS2 Spectrum processor provides de-isotoping but is not required. The MS Annika Detector identifies potential ion doublets, passes the spectra to the search node if doublets are found, and to a standard peptide identification algorithm if no doublets are found. In this case, we use the MS Amanda database search engine in conjunction with Percolator to identify linear peptides [9], [27]. Spectra with identified ion doublets are searched using the MS Annika Search node. The final combination of cross-link spectrum matches to cross-links as well as multilevel FDR control fall into the scope of the MS Annika Validator node. The optional xiView Exporter node can be used to export cross-links at different confidence levels, which can then be uploaded to xiView for evaluation.

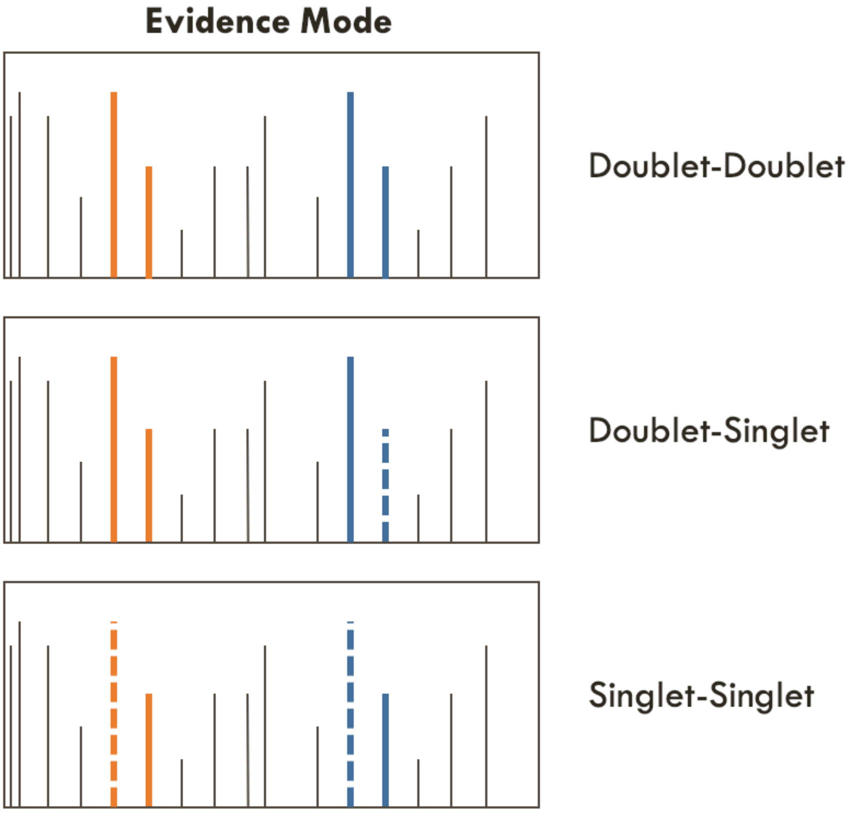

Supplementary Figure 2: Evidence Mode of MS Annika. In evidence mode the MS Annika Detector will detect the presence of a cross-link spectrum when a) both doublets are present (doublet-doublet), b) one double is present and the other one is only represented by a single peak (doublet- singlet) or c) the two cross-linked peptides are only represented by a single peak each (singlet-singlet). If ions are missing the MS Annika Detector recalculates them based on the measured precursor mass and on the used cross-linker.

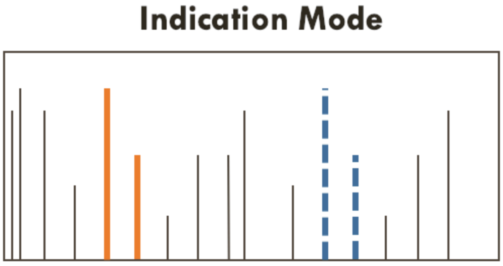

Supplementary Figure 3: Indication Mode of MS Annika. In indication mode the MS Annika Detector looks for doublet peaks with the appropriate distance only. The second doublet will be calculated based on the measured precursor mass and the used cross-linker.

			All Results		5% FDR		1% FDR		
Linker	PRIDE identifier	Comment	# CSMs	# Crosslinks	# CSMs	# Cross-links	# CSMs	# Cross-links	Refer-ence
DSSO	PXD010796	Human DB / with Background	28895	27059	551	45	382	39	[16]
DSSO	PXD010796	Human DB / no Background	23667	20819	931	98	725	67	[16]
DSAU	PXD018935	Fraction A	1566	820	606	159	488	119	[33]
DSAU	PXD018935	Fraction B	1166	672	423	157	344	133	[33]
BPD-NHP	PXD008975		198197	182211	9179	2651	6189	1874	[30]
DSBU	PXD012546	Fraction R1	180294	168290	3983	1902	2569	1353	[31]
DSBU	PXD014337		2421	1061	1613	284	1397	250	[20]
DSSO	PXD014337		2367	906	1605	228	1373	209	[20]
DSSO	PXD011861		13723	5908	5923	785	4814	632	[17]
DSSO	PXD022772	r1 & r2	1464	304	1188	183	1018	165	-
DSBU	PXD022772	r1 & r2	1472	342	1111	205	943	185	-
DSBSO	PXD016963		3146	913		224		163	[32]

Supplementary Table 1: Numbers of identified cross-links and CSMs for different data sets presented in this work. 1% and 5% FDR results are based on the FDR estimation in MS Annika.

**PXD014337, DSBU and DSSO**	*MS Annika*	*MeroX*	*XlinkX*	*pLink*
MS1 Tolerance	5 ppm	5 ppm	5 ppm	5 ppm
MS2 Tolerance	10 ppm	10 ppm	10 ppm	10 ppm
Enzyme	Trypsin	Trypsin	Trypsin	Trypsin
Missed Cleavages	3	3	3	3
Precursor offset	3	3	3	Not available
Cross-link modification on	K	K	K	K
PTMs	Carbamidomethyl (C, Static), Oxidation (M, Dynamic)	Carbamidomethyl (C, Static), Oxidation (M, Dynamic)	Carbamidomethyl (C, Static), Oxidation (M, Dynamic)	Carbamidomethyl (C, Static), Oxidation (M, Dynamic)
Search Mode	Combined Mode	RiseUp	MS2	MS-Cleavable

**PXD010796, with and without background**	*MS Annika*	*MeroX*	*XlinkX*
MS1 Tolerance	10 ppm	10 ppm	10 ppm
MS2 Tolerance	20 ppm	20 ppm	20 ppm
Enzyme	Trypsin	Trypsin	Trypsin
Missed Cleavages	4	3	4
Precursor offset	3	3	3
Cross-link modification on	K, N-Term	K, N-Term	K, N-Term
PTMs	Carbamidomethyl (C, Static), Oxidation (M, Dynamic)	Proteome-wide	Carbamidomethyl (C, Static), Oxidation (M, Dynamic)
Search Mode	Combined Mode	Proteome-wide mode	MS2_MS2

**PXD018935, DSAU**	*MS Annika*
MS1 Tolerance	4 ppm
MS2 Tolerance	8 ppm
Enzyme	Trypsin
Missed Cleavages	3

Precursor offset	5
Cross-link modification on	K, N-Term
PTMs	Carbamidomethyl (C, Static), Oxidation (M, Dynamic)
Search Mode	Combined Mode

**PXD008975, BDP-NHP**	*MS Annika*
MS1 Tolerance	10 ppm
MS2 Tolerance	20 ppm
Enzyme	Trypsin
Missed Cleavages	4
Precursor offset	5
Cross-link modification on	K, N-Term
PTMs	Carbamidomethyl (C, Static), Oxidation (M, Dynamic)
Search Mode	Combined Mode

**PXD012546, DSBU**	*MS Annika*
MS1 Tolerance	4 ppm
MS2 Tolerance	8 ppm
Enzyme	Trypsin
Missed Cleavages	3
Precursor offset	3
Cross-link modification on	K, N-Term
PTMs	Carbamidomethyl (C, Static), Oxidation (M, Dynamic)
Search Mode	Combined Mode

**PXD022772, Peplib DSSO/DSBU timsTOF**	*MS Annika*
MS1 Tolerance	5 ppm
MS2 Tolerance	10 ppm
Enzyme	Trypsin
Missed Cleavages	3
Precusor offset	3
Cross-link modification on	K, N-Term
PTMs	Carbamidomethyl (C, Static), Oxidation (M, Dynamic)
Search Mode	Combined Mode

**PXD016963, DSBSO**	*MS Annika*
MS1 Tolerance	10 ppm
MS2 Tolerance	20 ppm
Enzyme	Trypsin
Missed Cleavages	4
Precursor offset	5
Cross-link modification on	K, N-Term
PTMs	Carbamidomethyl (C, Static), Oxidation (M, Dynamic)
Search Mode	Combined Mode

Supplementary Table 2: Settings for different tools mentioned in the main text.

linker	Estimated CSM FDR	Calculated CSM FDR
DSBU (PXD014337, [20])	1%	1.45%
DSBU (PXD014337, [20])	5%	4.62%
DSSO (PXD014337, [20])	1%	2.50%
DSSO (PXD014337, [20])	5%	6.47%

Supplementary Table 3: CSM level FDRs for MS Annika. Comparison of calculated FDRs based on wrongly identified CSMs from different groups with estimated FDRs for 1% and 5% based on the data set of Beveridge and co-workers [20].

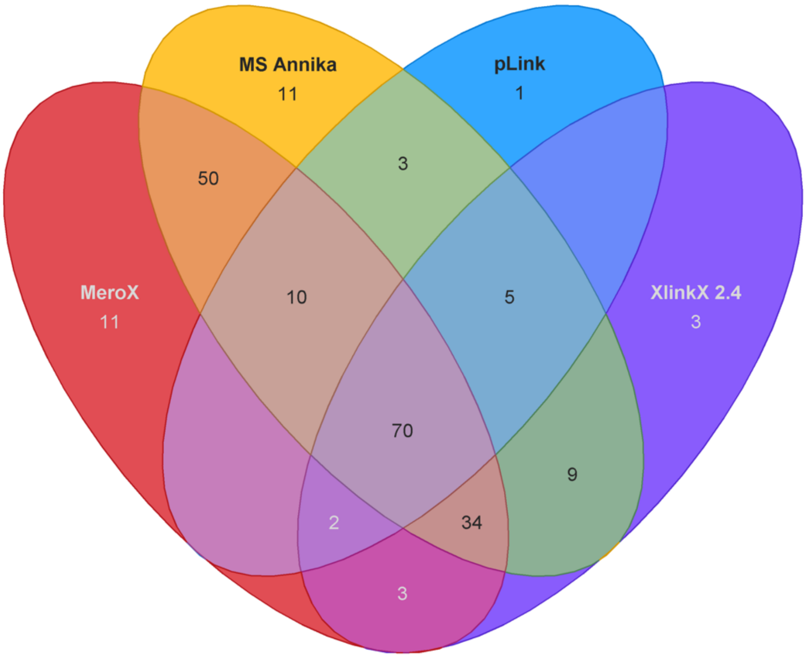

Supplementary Figure 4: Overlap of cross-linkers for DSSO linked peptides. Here the overlap of cross-links within the same group identified at 1% FDR of four different search engines, namely MS Annika, pLink, XLinkX, and Merox from the data set by Beveridge and co-workers are displayed [20].

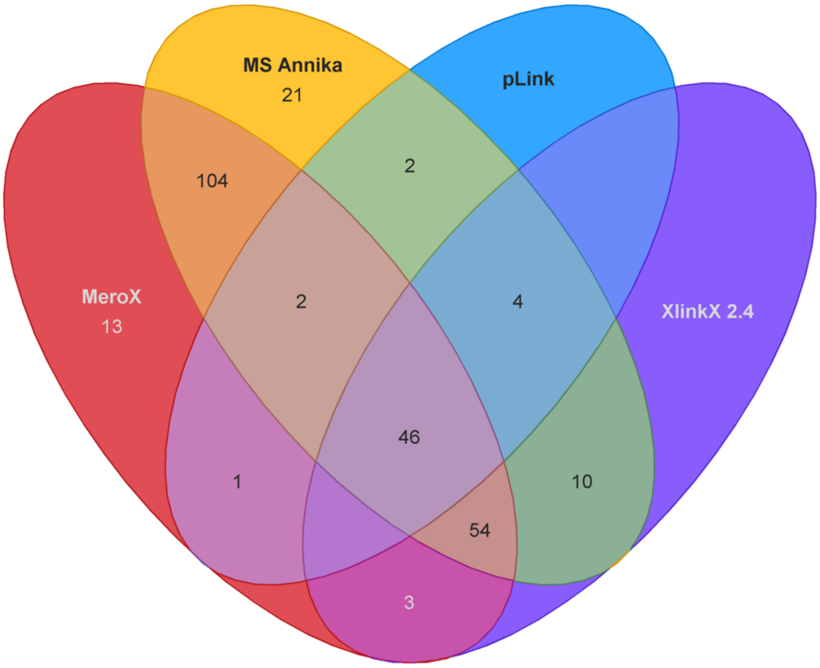

Supplementary Figure 5: Overlap of cross-linkers for DSBU linked peptides. Here the overlap of cross-links within the same group identified at 1% FDR of four different search engines, namely MS Annika, pLink, XLinkX, and MeroX from the data set by Beveridge and co-workers are displayed [20].

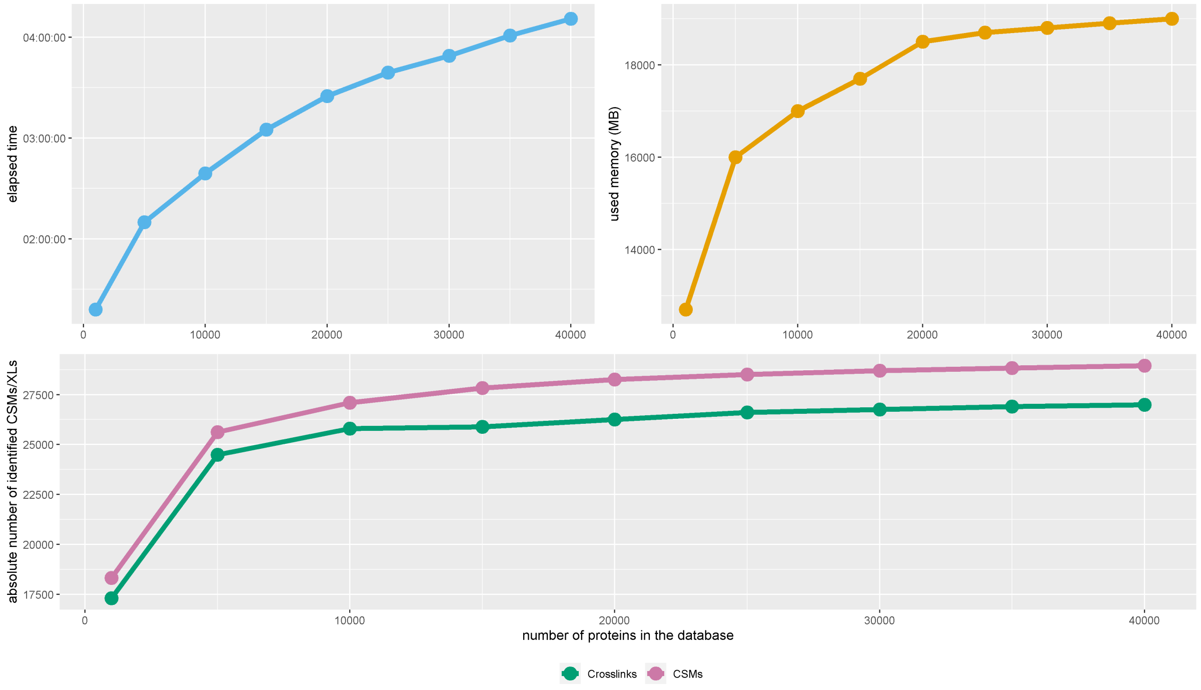

Supplementary Figure 6: Runtime and memory analysis. Randomly selected proteins have been sampled from a human uniprot database including isoforms and common contaminants. Resulting FASTA files ranged from 1000, 5000, to 40000 proteins. Runtimes and approximate memory consumptions are given in blue and orange on the top. On the bottom the total number of identified CSMs and cross-links (without applying any FDR threshold) are depicted to assess the data set size.

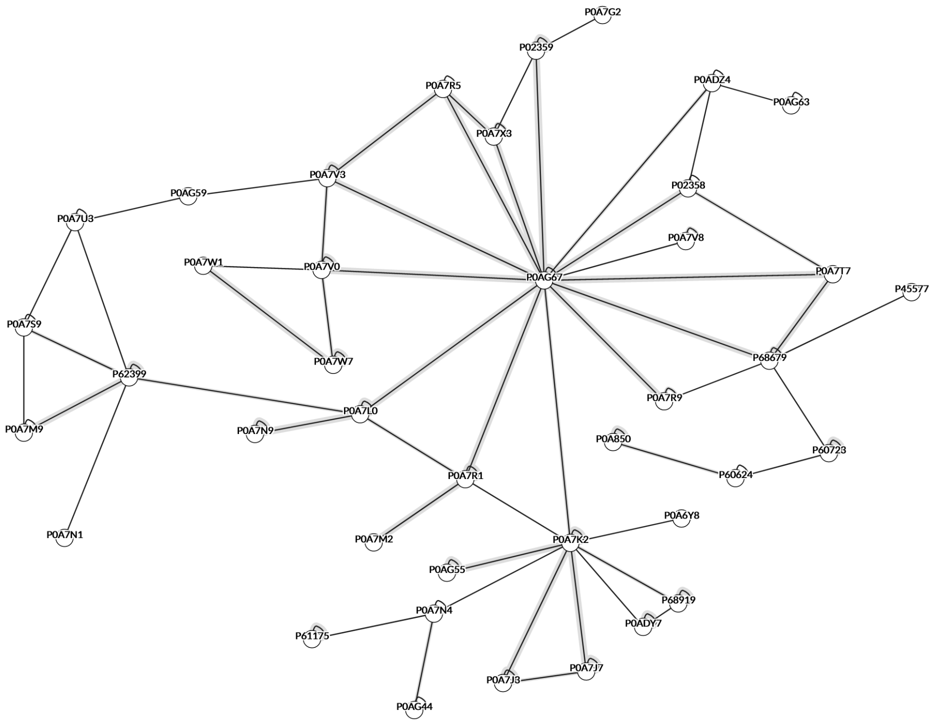

Supplementary Figure 7: Protein interaction network created within xiView, for cross-links that are within the 26 Angstrom distance constraints for DSSO. The protein interaction network was drawn from data published by Stieger and co-workers [2].

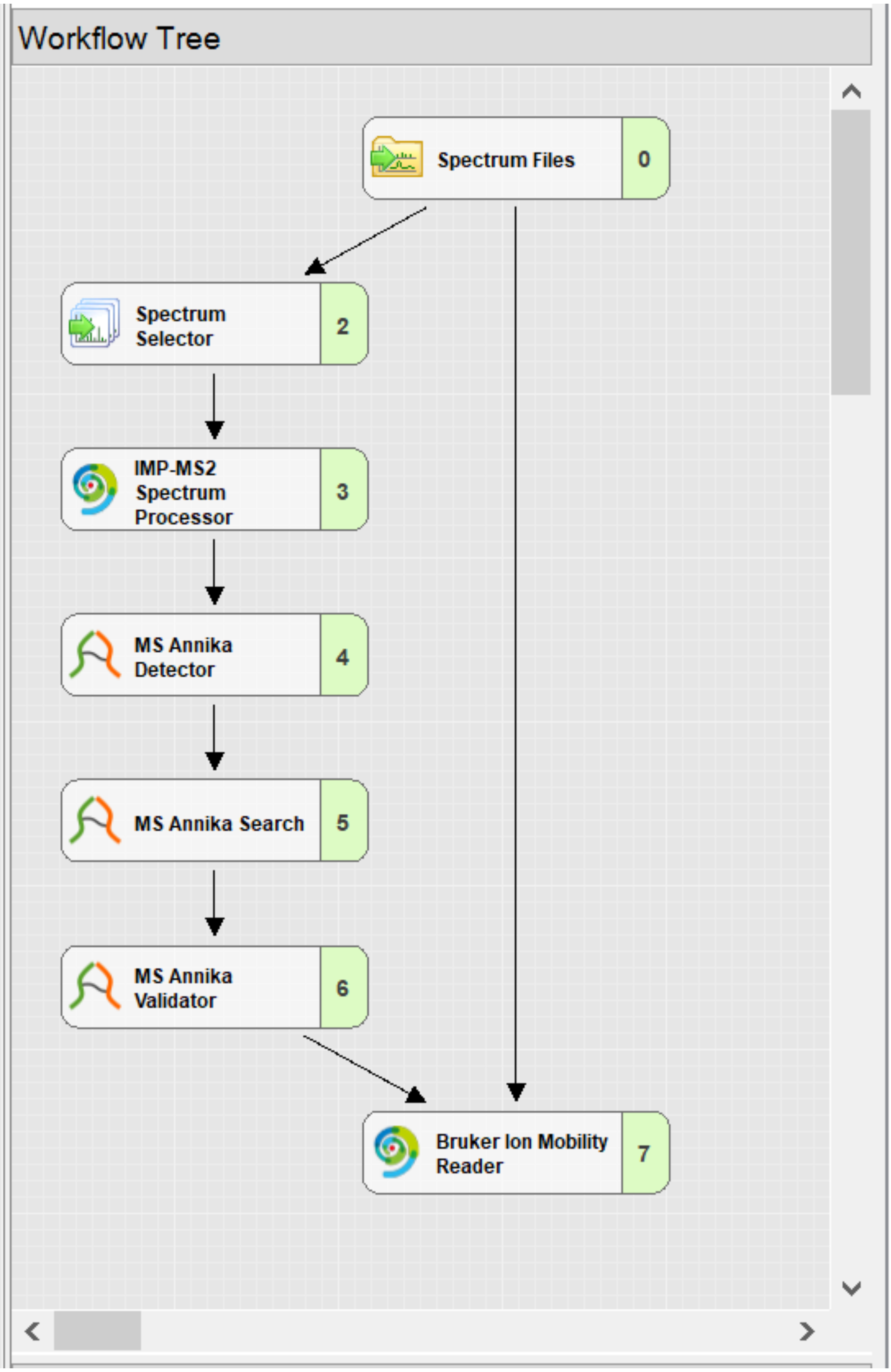

Supplementary Figure 8: Modified Workflow for timsTOF data, using the Bruker Ion Mobility Reader. This additional node can be installed during the setup process for MS Annika.

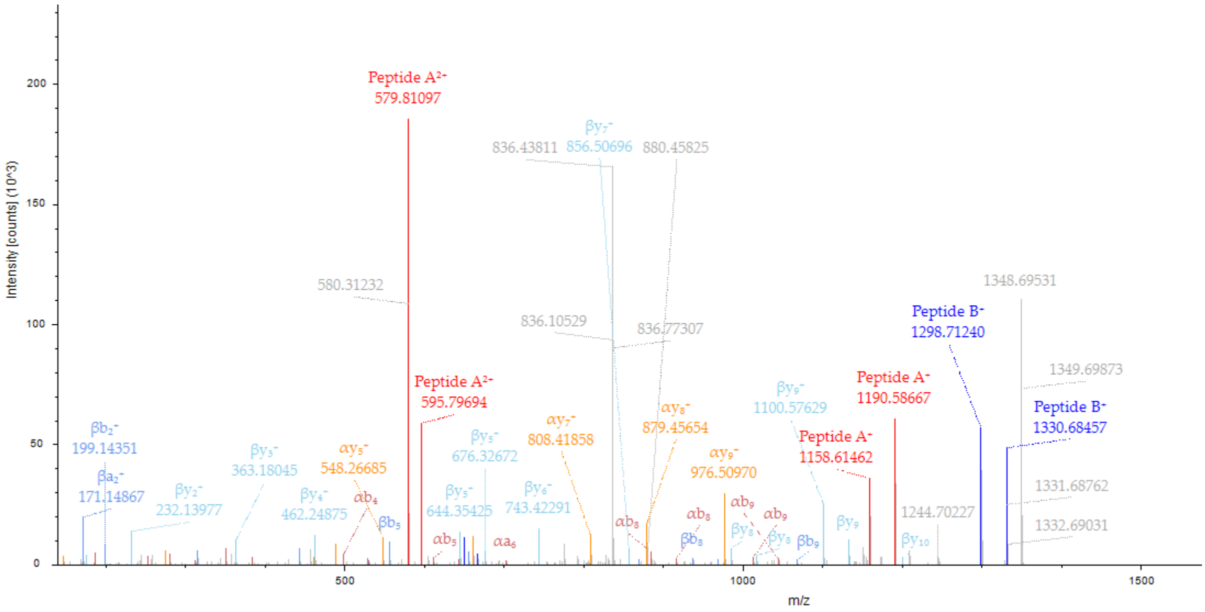

Supplementary Figure 9: *Example CSM of the DSSO data set by Beveridge et al. [20], as depicted in Thermo Proteome Discoverer 2.5. This spectrum represents scan number #20799 (z=+3, m/z = 836.10760) with the crosslinked peptides A (**K**PAFLSGEQK) and B (VVDELV**K**VMGR). Cross-linking positions are marked in bold. Peptides have been identified using Combined Mode, having a score of 660.3.*

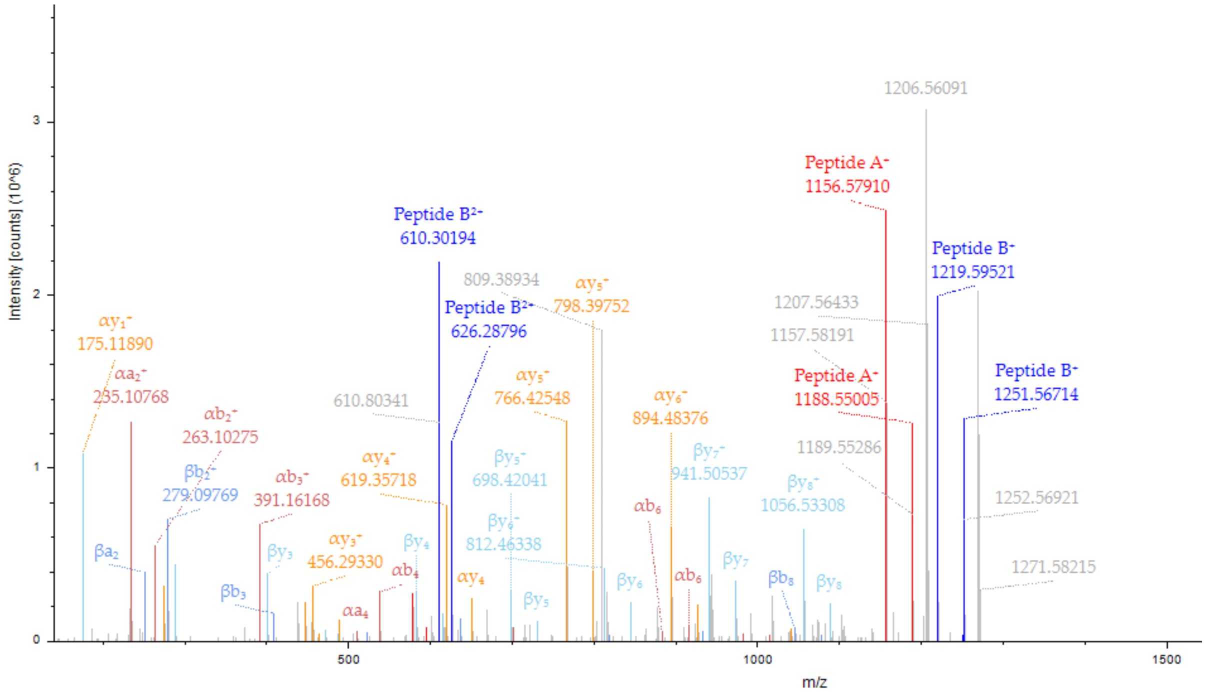

*Supplementary Figure 10: Example CSM of the DSSO data set by Beveridge et al. [20], as depicted in Thermo Proteome Discoverer 2.5. This spectrum represents scan number #20983 (z=+3, m/z = 809.0556) with the crosslinked peptides A (DFQFY**K**VR) and B (YDEND**K**LIR). Cross-linking positions are marked in bold. Peptides have been identified using Combined Mode, having a score of 465.9.*
