# Supplementary material for: MS Annika: A New Cross-Linking Search Engine
Source: J Proteome Res. 2021 Apr 14;20(5):2560–9. doi: 10.1021/acs.jproteome.0c01000 (PMC8155564; doi:10.1021/acs.jproteome.0c01000)
Supplement: Supplementary file 2 — pr0c01000_si_002.pdf [file pr0c01000_si_002.pdf]

Algorithm 1 $xlSpectra = DetectXLSpectra(allSpectra, crossLinker)$
Basic functionality of MS Annika Detector

```
Initialize xlSpectra as Map of spectra and List of MassPair
Initialize doubletMassDiff as difference between light and heavy part of crossLinker
for each spectrum s in allSpectra do
    Initialize candidateDoublets as List of Doublets
    Initialize validPairs as List of MassPairs
    Identify all Doublets in s based on doubletMassDiff and store in candidateDoublets
    if evidenceMode or combinedMode then
        for each d1 in candidateDoublets do
            for each d2 in candidateDoublets do
                if (light(d1) + heavy(d2) = precursor(s)) || (heavy(d1) + light(d2) = precursor(s)) then
                    Calculate peptide masses of d1 and d2 without attached linker fragment and store them
                    in validPairs
                end if
            end for
        end for
    end if
    if shotgunMode or combinedMode then
        for each d1 in candidateDoublets do
            Calculate light of d2 as precursor of s - heavy of d1
            Calculate peptide masses of d1 and d2 without attached linker fragment and store them in
            validPairs
        end for
    end if
    if size of validPairs > 0 then
        Remove duplicates in validPairs
        Store s and validPairs in xlSpectra
    end if
end for
```

Algorithm 2 $CSMs = IdentifyXLPeptides(xlSpectra, searchParameters)$
Basic functionality of MS Annika Search

Initialize $CSMs$ as List of results
for each (*spectrum*, $MassPairList$) in *xlSpectra* **do**
  Initialize *maxScore* with -1
  Initialize *bestPair* as Pair of *Peptide*
  for each *validPair* in $MassPairList$ **do**
    Initialize $peptideMassA$ as $firstMass(validPair)$
    Initialize $peptideMassB$ as $secondMass(validPair)$
    Identify best peptide hit using $MSAmandaIdentifySpectrum(spectrum, peptideMassA$ as precursor, $searchParameters)$ and store result in *scorePepA* and *peptideA*
    Initialize $peptideMassB$ as $secondMass(validPair)$
    Identify best peptide hit using $MSAmandaIdentifySpectrum(spectrum, peptideMassB$ as precursor, $searchParameters)$ and store result in *scorePepB* and *peptideB*
    Initialize *annikaScore* as $min(scorePepA, scorePepB)$
    if maxScore < *annikaScore* **then**
      Overwrite *maxScore* with *annikaScore* and store *peptideA* and *peptideB* as *bestPair*
    end if
  end for
  Store *maxScore* and *bestPair* in $CSMs$
end for

Algorithm 3 $(validatedCSMs, validatedXLs) = ValidateResults$
$(CSMs, CsmFDRHigh, CsmFDRMedium, XLFDRHigh, XLFDRMedium)$
Basic functionality of MS Annika Validator

Initialize $validatedCSMs$ as List of CSMs with an FDR Label {High — Middle — Low}
Initialize *validatedCrossLinks* as List of CrossLinks with an FDR Label {High — Middle — Low}
Merge CSMs to cross links based on their protein position and store them in *crossLinks*
for each *entry* in $CSMs$ **do**
  if isDecoy(*peptideA*(*entry*)) —— isDecoy(*peptideB*(*entry*)) **then**
    Mark *entry* as decoy hit
  end if
end for
Calculate FDR for thresholds $CsmFdrHigh$ and $CsmFdrMiddle$ in $CSMs$ and store results in $validatedCSMs$
for each *entry* in *crossLinks* **do**
  if isDecoy(*peptideA*(*entry*)) —— isDecoy(*peptideB*(*entry*)) **then**
    Mark *entry* as decoy hit
  end if
end for
Calculate FDR for thresholds $XLFdrHigh$ and $XLFdrMiddle$ in *crossLinks* and store results in *validatedCrossLinks*
